# Transfer of the Symbiotic Plasmid of *Rhizobium etli* CFN42 to Endophytic Bacteria Inside Nodules

**DOI:** 10.3389/fmicb.2020.01752

**Published:** 2020-07-29

**Authors:** Luis Alfredo Bañuelos-Vazquez, Daniel Cazares, Susana Rodríguez, Laura Cervantes-De la Luz, Rosana Sánchez-López, Lucas G. Castellani, Gonzalo Torres Tejerizo, Susana Brom

**Affiliations:** ^1^Programa de Ingeniería Genómica, Centro de Ciencias Genómicas, Universidad Nacional Autónoma de México, Cuernavaca, Mexico; ^2^Programa de Biología de Sistemas y Biología Sintética, Centro de Ciencias Genómicas, Universidad Nacional Autónoma de México, Cuernavaca, Mexico; ^3^Departamento de Biología Molecular de Plantas, Instituto de Biotecnología, Universidad Nacional Autónoma de México, Cuernavaca, Mexico; ^4^Departamento de Ciencias Biológicas, Facultad de Ciencias Exactas, Instituto de Biotecnología y Biología Molecular (IBBM) – CCT-CONICET-La Plata, Universidad Nacional de La Plata, La Plata, Argentina

**Keywords:** plasmid, conjugative transfer, nodulation, symbiosis, rhizobia

## Abstract

Conjugative transfer is one of the mechanisms allowing diversification and evolution of bacteria. *Rhizobium etli* CFN42 is a bacterial strain whose habitat is the rhizosphere and is able to form nodules as a result of the nitrogen-fixing symbiotic relationship it may establish with the roots of *Phaseolus vulgaris*. *R. etli* CFN42 contains one chromosome and six large plasmids (pRet42a – pRet42f). Most of the genetic information involved in the establishment of the symbiosis is localized on plasmid pRet42d, named as the symbiotic plasmid (pSym). This plasmid is able to perform conjugation, using pSym encoded transfer genes controlled by the RctA/RctB system. Another plasmid of CFN42, pRet42a, has been shown to perform conjugative transfer not only *in vitro*, but also on the surface of roots and inside nodules, using other rhizobia as recipients. In addition to the rhizobia involved in the formation of nodules, these structures have been shown to contain endophytic bacteria from different genera and species. In this work, we have explored the conjugative transfer of the pSym (pRet42d) from *R. etli* CFN42 to endophytic bacteria as putative recipients, using as donor a CFN42 derivative labeled with GFP in the pRet42d and RFP in the chromosome. We were able to isolate some transconjugants, which inherit the GFP, but not the RFP marker. Some of them were identified, analyzed and evaluated for their ability to nodulate. We found transconjugants from genera such as *Stenotrophomonas*, *Achromobacter*, and *Bacillus*, among others. Although all the transconjugants carried the GFP marker, and *nod, fix*, and *nif* genes from pRet42d, not all were able to nodulate. Ultrastructure microscopy analysis showed some differences in the structure of the nodules of one of the transconjugants. A replicon of the size of pRet42d (371 Kb) could not be visualized in the transconjugants, suggesting that the pSym or a segment of the plasmid is integrated in the chromosome of the recipients. These findings strengthen the proposal that nodules constitute a propitious environment for exchange of genetic information among bacteria, in addition to their function as structures where nitrogen fixation and assimilation takes place.

## Introduction

*Rhizobium etli* CFN42 is able to establish a symbiotic relationship with the roots of *Phaseolus vulgaris* plants, forming nitrogen-fixing nodules. The ability to nodulate and fix nitrogen in association with leguminous plants is a characteristic shared by many rhizobial strains (Gage, 2004; Wielbo, 2012; Quinto et al., 2014; Westhoek et al., 2017). This ability depends on the presence of a set of genes required for nodulation (nod) and nitrogen fixation (fix and nif). In many strains, the *nod, fix*, and *nif* genes are localized on plasmids (pSym) or on symbiotic islands (Romero and Brom, 2004; González et al., 2010). Some plasmids and genomic islands are known for their capacity to carry out horizontal transfer by conjugation. Symbiotic and non-symbiotic plasmids from diverse rhizobial strains have been shown to contain DNA transfer and replication (Dtr) and mating pair formation (Mpf) genes, involved in conjugative transfer (Ding and Hynes, 2009; Bañuelos-Vazquez et al., 2017). Studies have been done regarding the regulation mechanism of rhizobial plasmids. To date four main types have been described. The most prevalent is regulation by quorum-sensing (type I), the second most distributed mechanism is through regulation by the RctA repressor (type II). Analysis of TraA and TraG sequence phylogeny led to the proposal of type III and type IV systems (Ding and Hynes, 2009; Ding et al., 2013; Torres Tejerizo et al., 2014; Wathugala et al., 2020).

*R. etli* CFN42 contains one chromosome and six plasmids, named pRet42a to pRet42f, whose sizes range between 184 and 642 Kb (González et al., 2006). Plasmid pRet42d corresponds to the pSym. This plasmid is able to transfer by conjugation using two different mechanisms. The first system allowing transfer of pRet42d depends on its cointegration with plasmid pRet42a. pRet42a is a conjugative plasmid that contains Dtr and Mpf genes whose expression is regulated by quorum-sensing (Tun-Garrido et al., 2003). Plasmids pRet42a and pRet42d can form cointegrates through homologous RecA-dependent recombination, or through a site-specific recombination system. The cointegrate transfers using the pRet42a-encoded transfer genes. In the recipient, the cointegrate resolves regenerating the wild-type plasmids in 90% of the events, while in 10% of the ransconjugants the resolution of the cointegrate leads to the formation of recombinant plasmids (Brom et al., 2004). The second transfer mechanism depends on Dtr and Mpf genes encoded in pRet42d. These genes are constitutively repressed by the RctA regulator. Only when RctA is inactivated by mutation, or the positive regulator RctB is overexpressed, the Dtr and Mpf genes are activated and transfer occurs (Pérez-Mendoza et al., 2005; Nogales et al., 2013). This system is also present in pSymA of *Sinorhizobium meliloti*, where the regulation has some differences compared to that of *R. etli*. Bioinformatics has shown that *rctA* and *rctB* are also present in the pAT plasmid of *Agrobacterium tumefaciens*, where they are under quorum-sensing control (reviewed in Bañuelos-Vazquez et al., 2017). The natural conditions that allow expression of this system are as yet unknown.

Recently, we have determined that conjugative transfer of pRet42a from *R. etli* CFN42 occurs on the root surface and also inside bean nodules (Bañuelos-Vazquez et al., 2019). The experiments were done introducing recipient and donor strains, but in a control where only the donor was introduced, we also found some putative transconjugants, and speculated that they may be the result of plasmid transfer to endophytic recipient bacteria residing in the nodules. Nodules often contain a great variety of bacterial populations from different genera and species, such as *Agrobacterium*, *Enterobacter*, *Chryseobacterium*, *Sphingobacterium*, *Actinobacteria*, and unclassified *Enterobacteriaceae.* The relative abundance of the different bacteria seems to depend on the soil and plant types. For example, *Enterobacter* spp., *Pseudomonas* spp., and *Bacillus* spp. were found to be the most abundant endophytic bacteria in some legumes (Dudeja et al., 2012; De Meyer et al., 2015; Leite et al., 2016; Lu et al., 2017). In general, these bacteria are considered to be beneficial for the plant, either through biological nitrogen fixation, or plant growth promotion (Dudeja et al., 2012), however, there is no information available regarding genetic interactions among the nodule endophytes. The aim of this work was to determine if the pSym of *R. etli* CFN42 is able to transfer to endophytic bacteria in the nodule, using the RctA-regulated transfer genes that it encodes.

## Materials and Methods

### Bacterial Strains and Plasmids

The bacterial strains and plasmids used in this work are described in Table 1. *Rhizobium etli* strains were grown on PY medium at 30°C (supplemented with 10 mM CaCl_2_) (Noel et al., 1984). Endophytic bacteria were grown on *Pseudomonas* Isolation Agar (PIA) (Catalog No. 292710) and *Pseudomonas* Agar Pyocyanin (PAP) (Catalog No. 244910), were purchased from DIFCO. Antibiotics were added at the following concentrations (in μg/ml); Nalidixic Acid (Nal) 20, Gentamicin (Gm) 30, and Spectinomycin (Sp) 100.

**TABLE 1:** Strains and plasmids used in this work.

**Strains and plasmids**	**Relevant characteristics**	**Features**	**References**
***R. etli***			
CFNX182	Derivative of CFN42, cured of pRet42a	Nal	Brom et al., 1992
CFNX182-1	Derivative of CFNX182 with RFP in the chromosome and GFP and SpR in pRet42d	Nal, Gm, Sp, RFP and GFP	This work
**TER**			
TER22	Strain isolated from nodules infected with CFNX182-1	Sp, GFP	This work
TER23	Strain isolated from nodules infected with CFNX182-1	Sp, GFP	This work
TER31	Strain isolated from nodules infected with CFNX182-1	Sp, GFP	This work
TER33	Strain isolated from nodules infected with CFNX182-1	Sp, GFP	This work
TER38	Strain isolated from nodules infected with CFNX182-1	Sp, GFP	This work
TER39	Strain isolated from nodules infected with CFNX182-1	Sp, GFP	This work
TER40	Strain isolated from nodules infected with CFNX182-1	Sp, GFP	This work
TER49	Strain isolated from nodules infected with CFNX182-1	Sp, GFP	This work
TER55	Strain isolated from nodules infected with CFNX182-1	Sp, GFP	This work
***E. coli***			
DH5α	supE44 ΔlacU169 (Φ80lacZΔM15) hsdR17 recA1 endA1 gyrA96 thi-1 relA1	Nal	Sambrok et al., 1989
S17-1	E. coli 294:[RP4-2 (Tc:Mu) (Km:Tn7)] thi pro hsdR hsdM ΔrecA	Km	Simon, 1984
**Plasmids**			
pK18mob	High copy number cloning vector	Km	Schäfer et al., 1994
pK18mob-sacB	Cloning vector, mobilizable	Km	Schäfer et al., 1994
pCR^TM^2.1-TOPO^TM^	Cloning vector	Km, Amp	Invitrogen^TM^
pGX33	Derivative of pCR^TM^2.1-TOPO^TM^ with a 579 pb fragment from pRet42d	Km, Amp	This work
pGX77	Derivative of pK18mob-SacB harboring a pRet42d-fragment from pGX33 (cloned as a *Pst*I and *Hind*III fragment)	Km	This work
pGX537	Vector that carries a 4.1 Kbp-*Not*I cassette (promoter NptII -gfpmut3^∗^- ΩSp)	Km, Sp	Torres Tejerizo et al., 2015
pGX542	Derivative of pGX77 harboring the *Not*I-cassette from pGX537.	Km, Sp	This work

### Plant Growth Conditions

*P. vulgaris* cv Negro Jamapa seeds were surface-sterilized in 96% (v/v) ethanol for 30 sec, then incubated for seven min in 10% (v/v) commercial sodium hypochlorite, and finally rinsed five times in sterile distilled water. Subsequently, seeds were germinated on moist sterile paper towels at 30°C for 2 days in darkness (Cárdenas et al., 1995; Zepeda et al., 2014).

Selected uniform seedlings were planted in CYG^TM^ seed germination pouches under controlled environmental conditions. Each seedling was inoculated with 1 ml of a culture of the desired strain at 0.05 OD at 600 nm and grown in a controlled environmental chamber (14/10 h light/dark cycle, 22/16°C and relative humidity 60–70%). Each germination pouch was watered with 10 ml of Fahraeus nitrogen-free nutrient solution (Fahraeus, 1957).

Alternatively, seedlings were planted in hydroponic trays containing eight L of Fahraeus (Fahraeus, 1957) nitrogen- free nutrient solution, and each seedling was inoculated with 1 ml of liquid culture of the desired strain.

### Tagging of pRet42d and Chromosome of *R. etli* CFN42 With GFP and RFP

First, we tagged the chromosome of CFNX182 (a derivative of *R. etli* CFN42 cured of pRet42a) by means of a Mini-Tn*7*-DsRed cassette. Mini-Tn*7* integration occurs downstream of the gene encoding glucosamine-6-phosphate synthetase (*glmS*) (Lambertsen et al., 2004), generating CFNX182-RFP.

Plasmid pRet42d from CFNX182-RFP was tagged with GFP as follows: first we amplified a fragment of 579 bp (primers p42d_left_in and p42d_right_in) and cloned it in pCR^TM^2.1-TOPO^TM^, generating plasmid pGX33. The 579 bp fragment is located between genes RHE_PD00110 andRHE_PD00113, which are arranged tail to tail, and does not interrupt any open reading frame. Plasmid pGX33 was further digested with *Pst*I and *Hin*dIII. The released 655 bp fragment was cloned into the pK18mob-sacB vector previously digested with *Pst*I and *Hin*dIII, generating plasmid pGX77. Next, we introduced a *Not*I-cassette with promoter *nptII*-*gfp*mut3^∗^-Sp from plasmid pGX537 (Torres Tejerizo et al., 2015) into the *Not*I site of pGX77. The resulting vector, pGX542, was introduced into *R. etli* CFNX182-RFP and recombination events were selected, generating CFNX182-1, a CFN42 derivative lacking pRet42a, with RFP in the chromosome and GFP in pRet42d. The correctness of the integration was evaluated by PCR, using primers internal to the cassette CasNot-Ter-out and Pneo-out and external primers from pRet42d for each side, p42d_left_out and p42d_right_out. The oligonucleotides used in this work are shown in Supplementary Table S1.

### Bacterial Matings in Symbiotic Conditions

Two-day-old germinated seedlings were introduced into tubes with Fahraeus medium (Fahraeus, 1957) inoculated with the donor strain (CFNX182-1) at 0.05 OD at 600 nm. After 21 days the nodules were processed to recover putative transconjugants.

### Isolation of Culturable Endophytic Bacteria Containing the GFP Labeled pRet42d From Root Nodules of *P. vulgaris*

The endophytic bacteria containing the pRet42d-GFP were recovered from nodules (about 150), collected from three *P. vulgaris* plants. To isolate bacteria from the nodules, the nodules were collected and sterilized with 10% (v/v) commercial sodium hypochlorite, and rinsed five times in sterile distilled water. The sterilized nodules were crushed in 1 ml of ice-cold buffer containing 0.25 M mannitol, 0–05 M Tris-HCl, pH 7.8 and 100 mg polyvynyl-polypirrolne (PVPP). To remove all the plant tissue material, the suspension was centrifuged at 3000 rpm for 10 min at 4°C. Supernatant was carefully removed and centrifuged again at 3000 rpm at 4°C for 4 min. After removing the supernatant, the pellet (containing bacteria and bacteroids) was washed and resuspended in 1 ml PBS buffer (Tsyganov et al., 2003), and then plated on calcium-free PY, PIA, and PAP media supplemented with Sp 100. These media present conditions not favorable for the growth of the donor strain CFNX182-1, increasing the possibility of recovering transconjugants where the plasmid was transferred to endophytic recipients. After 48 h of incubation (30°C) the colonies were analyzed for green fluorescence at a wavelength of 488 nm and the fluorescent colonies were purified.

### PCR Amplification of 16S rRNA Sequences and Genes Involved in Nodulation and Nitrogen Fixation in the Putative Transconjugants of Endophytic Recipient (TER) Strains

Putative TER strains recovered from nodules of *P*. *vulgaris* were classified at the genus level by phylogenetic analysis of the 16S rRNA gene (*rrs*) sequences amplified with the universal fD1 and rD1 primers (Supplementary Table S1). To analyze the presence of sequences from the pSym of *R. etli* CFN42 in the TER strains, PCR amplifications were performed with primers from nodulation genes: *nodA* and *nodD1*, and nitrogen fixation genes: *fixNd* and *nifH* (Supplementary Table S1), using DNA from the TER strains as template.

### Taxonomic Identification of Putative TER Strains

The taxonomic identification of TER candidates at genera level was performed only for the strains that produced PCR products with primers for the nodulation and nitrogen fixation genes in addition to the GFP gene. The 16S rRNA gene from these strains was amplified by PCR from their genomic DNA with the universal primers fD1 and rD1 using standard conditions (Supplementary Table S1). The amplicons were purified and then sequenced by Sanger technology in the USSDNA, IBt-UNAM. The bacterial genera were determined by performing a BLASTn of the obtained sequences against the NCBI database in order to identify the genera of the closest matches. The homologous sequences (only type strains were used as reference) were aligned (from 800 nucleotides of the internal part of the sequence) with Clustal Omega (Sievers and Higgins, 2013) and this alignment was used to generate a Maximum-likelihood tree with PhyML 3.0 (Guindon et al., 2010) to visualize the phylogenetic associations of TER strains.

### Stability Assay

A single colony of each strain was picked from a selective plate, inoculated into 5 ml of PY medium with antibiotic (Sp 100), and incubated overnight at 30°C. After that, the culture was washed and inoculated in a flask with 50 ml of PY medium at 0.05 OD, without antibiotic. Samples were collected at inoculation (T0) and after 72 h of incubation (T72) and plated on media with and without the corresponding antibiotics.

### Nitrogenase Activity and Leghemoglobin Content

After 21 days of inoculation, eight plants grown in hydroponic trays were harvested for nitrogenase activity determined by acetylene reduction activity (ARA), using a gas chromatograph equipped with a flame ionization detector and a capillary column (Varian 3300 Gas Chromatograph), as described by Hardy et al. (1968). Specific activity is expressed as μmol ethylene h^–1^ per gram of nodule dry weight (NDW). Nodule, plant and root dry weight were determined after drying at 60°C for 2 days.

Leghemoglobin content was measured as previously described by LaRue and Child (1979), from eight plants grown in seed germination pouches. Nodules (0.3 g) were ground with 4 ml LB extraction buffer (40 mM Na_2_HPO_4_. 2H_2_O, pH 7.4; 10 mM NaH_2_PO_4_. H_2_O, pH 7.4; 0.02% K_3_Fe (CN)_6_; 0.1% NaHCO_3_) supplemented with 0.1 g polyvinylpolyrrolidone (PVPP). The homogenate was centrifuged at 12,000 rpm at 4°C for 20 min. Clear supernatant (50 μl) and saturated oxalic acid (3 ml) were mixed in screw-capped tubes, which were sealed and autoclaved for 30 min at 120°C and then allowed to cool at room temperature. The fluorescence of the solutions was measured using a microplate reader (Synergy^TM^ H1). The excitation wavelength was 405 nm and the emission was 650 nm. The difference in fluorescence between heated and unheated samples is proportional to haem protein content. These experiments were done with plants grown in seed germination pouches.

### Microscopy and Flow Cytometry Analysis

For preparation of the samples, and analysis by microscopy of fluorescence and flow cytometry, we used the protocol described by Bañuelos-Vazquez et al. (2019). Briefly, for flow cytometry analysis, hydroponic tubes containing *P. vulgaris* seedlings were inoculated at 0.05 OD at 600 nm only with CFNX182-1 as a donor and no recipient strain. For the analysis we used three biological and three technical replicates. After 21 dpi, bacteria were isolated from the medium by centrifugation, and from the root surface by subjecting them to ultrasound for 20 min in a Branson 200 ultrasonic cleaner. After taking out the roots, the medium was centrifuged for 15 min at 5000 rpm, at 4°C to recover the bacteria attached to the roots surface. Bacteria were recovered from sterilized nodules (about 150) using the protocol from Tsyganov et al. (2003). Finally, bacteria from the different sources were resuspended in 500 μl of PBS buffer. For each sample, images were simultaneously collected for 20,000 events. Acquisition was performed on an Imaging flow cytometer (ImageStream^x^; Amnis/EMD Millipore, Seattle, WA), and the Analysis was performed with IDEAS^®^ software version 5.0, and individual cell images were created using IDEAS^®^ software version 6.1 (Amnis Corp., Seattle, WA). All experiments were repeated at least three times, their average and standard deviations are shown. For microscopy of fluorescence analysis, we used an Olympus IX-81 inverted microscope (Tokyo, Japan) described in Bañuelos-Vazquez et al. (2019). Images were processed using ImageJ 1.47v (Wayne Rasband National Institutes of Health) and Adobe Photoshop 7.0 software (Adobe Systems, Mountain View, CA).

### Histological and Transmission Electron Microscopy Analysis

Nodule samples, harvested at 21 days post inoculation, were fixed in 2.5% glutaraldehyde, 4% paraformaldehyde in 0.1 M Na-cacodylate buffer (pH7.2), post-fixed with 1% osmium tetroxide, and dehydrated with graded series of ethanol (10–100%), as described in Sánchez-López et al. (2011). Samples were gradually embedded in EMbed 812 resin and sectioned using an ultramicrotome (Ultracut Leica). Semi-thin sections (0.5–1.0 mm) were stained with 0.1% toluidine blue and examined with an AX10 microscope coupled to Axiocam 503 color (Zeiss). Ultra-thin sections were stained with 2% uranyl acetate and observed in a Transmission Electron Microscope Libra 120 Plus (ZEISS) operated at 70 kV. Image processing was performed using Fiji ImageJ package.

## Results

### Identification and Characterization of Transconjugants Generated by Transfer of pRet42d to Endophytic Nodule Bacteria

**FIGURE 1:**
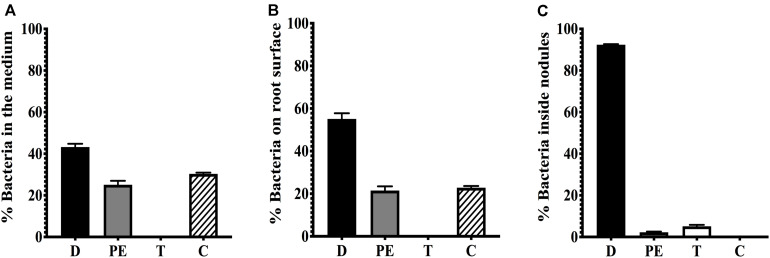
Flow cytometry analysis of bacteria recovered from the root surface and nodules of plants infected with CFNX182-1. Plants were inoculated with strain CFNX182-1, which carries a RFP marker on the chromosome and a GFP marker on the pSym. Bacteria were recovered at 20 dpi from: **(A)** the medium, **(B)** the root surface and **(C)** nodules. Populations corresponding to donors (D) show red plus green fluorescence, putative endophytic recipients (PE) have no fluorescence, transconjugants of endophytic recipients (T) carry GFP and show green fluorescence, and pSym-cured derivatives (C) with RFP show red fluorescence.

**FIGURE 2:**
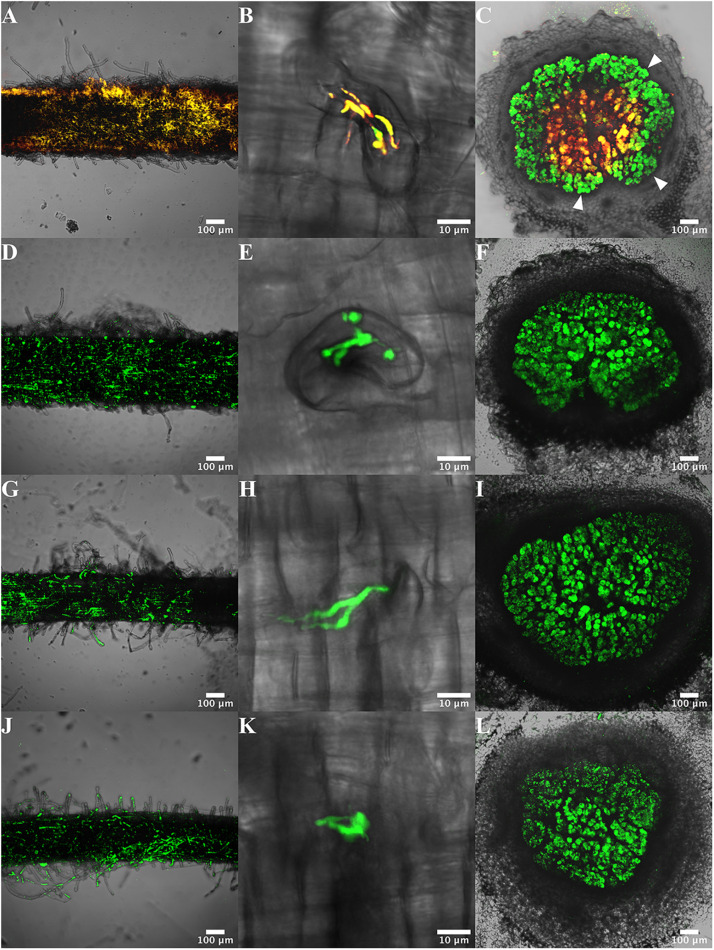
Visualization of roots, infection threads and nodules of *P. vulgaris* plants infected with CFNX182-1 and TER strains. Plants were inoculated with the donor strain CFNX182-1 **(A–C)**, and with transconjugant strains TER31 **(D–F)**, TER38 **(G–I)** and TER49 **(J–L)**. Samples of the root surface **(A,D,G,J)**, infection thread **(B,E,H,K),** and nodules **(C,F,I,L)** were analyzed 21 dpi by confocal microscopy. CFNX182-1 is yellow (red plus green fluorescence) and transconjugants of endophytic recipients (TER) carry green fluorescence. Arrowheads indicate the localization of transconjugants **(C)**.

To determine if the conjugative transfer of pSym to endophytic bacteria of root nodules occurs, we used as donor strain CFNX182-1, a derivative of *R. etli* CFN42 cured of pRet42a, with a GFP marker in the symbiotic plasmid and an RFP marker in the chromosome. *P. vulgaris* roots were inoculated with this strain as a donor, without adding a recipient strain, in hydroponic conditions. At 21 days post inoculation (dpi), we recovered the bacteria from the nodules as described in section “Materials and Methods”. To have a quantitative measure of the pSym transfer we analyzed the distribution of the bacterial population collected from either the medium where the plants were grown, the root surface and nodules by flow cytometry. Putative transconjugants acquire the GFP fluorescence located in the plasmid, but not the RFP fluorescence associated to the chromosome of the donor strain. The results showed that there were no transconjugants in the medium or on the root surface but, interestingly, some transconjugants were present in the nodules (Figure 1). Analysis by confocal microscopy confirmed that transconjugants were present only in nodules but not on the root surface or infection threads (Figures 2A–C). Fifty-five strains that showed green fluorescence were isolated from approximately 150 nodules, collected from three plants, as described in section “Materials and Methods”. The use of media supplemented with spectinomycin and detection of fluorescent colonies represented the selection criteria applied to skew the isolation toward transconjugants of endophytic recipients (TER) carrying the symbiotic plasmid pRet42d. Different specific *R. etli* genes involved in nodulation (*nodA* and *nodD1*) and nitrogen fixation (*fixNd* and *nifH*) of the *R. etli* pSym, as well as the GFP coding sequence, were selected to determine their presence in the TER strains. The results (Supplementary Figure S1 and Table 2) showed that all the markers were present in only 9 of the 55 isolates, suggesting that they contain the pRet42d, or a fragment of the plasmid. These 9 endophytic isolates were cultured and genomic DNA extraction was performed as described in section “Materials and Methods”. The DNAs obtained were used to amplify the internal fragments of the 16S gene by PCR. The amplification products obtained were purified and sequenced by Sanger technology at the Institute of Biotechnology, UNAM (Supplementary Table S2).

**TABLE 2:** Characteristics of putative transconjugants from endophytic recipients isolated from nodules.

**Name**	**Genus^a^**	**Class**	**Coverage –identity^b^**	**nodD1^c^**	**nodA^c^**	**fixNd^c^**	**nifH^c^**	**GFP^c^**	**Nodulation^d^**
TER22	*Brevundimonas* sp.	Alphaproteobacteria	100–99.88%	+	+	+	+	+	+
TER23	*Pseudomonas* sp.	Gammaproteobacteria	100–100%	+	+	+	+	+	−
TER31	*Stenotrophomonas* sp.	Gammaproteobacteria	100–94.16%	+	+	+	+	+	+
TER33	*Bacillus* sp.	Firmicutes-Bacilli	100–99.88	+	+	+	+	+	−
TER38	*Achromobacter* sp.	Betaproteobacteria	100–99.25%	+	+	+	+	+	+
TER39	*Ochrobactrum* sp.	Alphaproteobacteria	100–100%	+	+	+	+	+	−
TER40	*Mesorhizobium* sp.	Alphaproteobacteria	99–98.75%	+	+	+	+	+	−
TER49	*Phyllobacterium* sp.	Alphaproteobacteria	100–98.64%	+	+	+	+	+	+
TER55	*Achromobacter* sp.	Betaproteobacteria	99–98.61%	+	+	+	+	+	−

*^*a*^Genera were identified by sequencing PCR products obtained with 16S primers, using DNA of each strain as template. ^*b*^The coverage and identity were determined by BLASTn of the internal fragments of the 16s RNA. ^*c*^Presence of R. etli CFNX182-1 pSym sequence was determined by the obtention of PCR products of the different genes, using DNA of each strain as template. ^*d*^Nodulation ability was determined by inoculating bean seedlings with cultures of each purified strain, and determining the presence of nodules at 20 dpi.*

The sequences obtained were used to perform a homolog search (with the BLASTn tool of the NCBI database). The position of the TER strains in the phylogenetic tree showed that they present a wide taxonomic diversity, belonging to eight different genera: Four were alpha-proteobacteria: *Mesorhizobium*, *Brevundimonas*, *Phyllobacterium*, *Ochrobactrum*, one was a beta- proteobacteria: *Achromobacter*, two were gamma-proteobacteria: *Pseudomonas* and *Stenotrophomonas*, and one was from the Firmicutes phylum: *Bacillus* (Table 2, Supplementary Table S2, and Supplementary Figure S2).

### Stability of the Transferred pRet42d Genetic Marker in the Transconjugants

Stability assays of the TER strains (see Section “Materials and Methods”) showed that the antibiotic resistance marker associated to pRet42d was very stable after 72 h of inoculation (Supplementary Figure S3). This could be due to two possibilities: first, that the symbiotic plasmid (pSym) has been integrated into the chromosome of the TER strain, maybe due to incompatibility with an endogenous plasmid and therefore the effect on its stability, and second, that the *Rhizobium etli* pSym is quite compatible with the endophytic strains. We favor the first possibility, as we could not identify a plasmid of the size of the pSym in the TER strains using Eckhardt type gels (Eckhardt, 1978), which allow the visualization of large plasmids (data not shown).

### Some TER Strains Are Able to Nodulate and Fix Nitrogen in *Phaseolus vulgaris* Roots

In order to explore whether TER strains formed effective nitrogen-fixing nodules in common bean roots, an experiment was performed as follows: 2-days-post-germination (dpg) *P. vulgaris* seedlings were independently inoculated with each of the 9 purified TER strains that presented *nod*, *fix, nif* and GFP genes, using also donor strain CFNX182-1 as a control. Only four of the strains: TER22, TER31, TER38, and TER49 were able to produce nodules in *P*. *vulgaris* roots at 21 dpi. For all experiments, uninoculated controls did not contain nodules. For strain TER31, bacteria were isolated from approximately 300 nodules from 3 plants. The nodules were surface sterilized and crushed, and bacteria were recovered in PY medium with calcium and spectinomycin (3.15 × 10^3^), and in PY without calcium but with spectinomycin (3.4 × 10^3^). *Rhizobium* CFNX182-1 does not grow without calcium, assuring that all recovered bacteria correspond to the inoculated strain TER31, and there is no contamination by *R. etli.*

**FIGURE 3:**
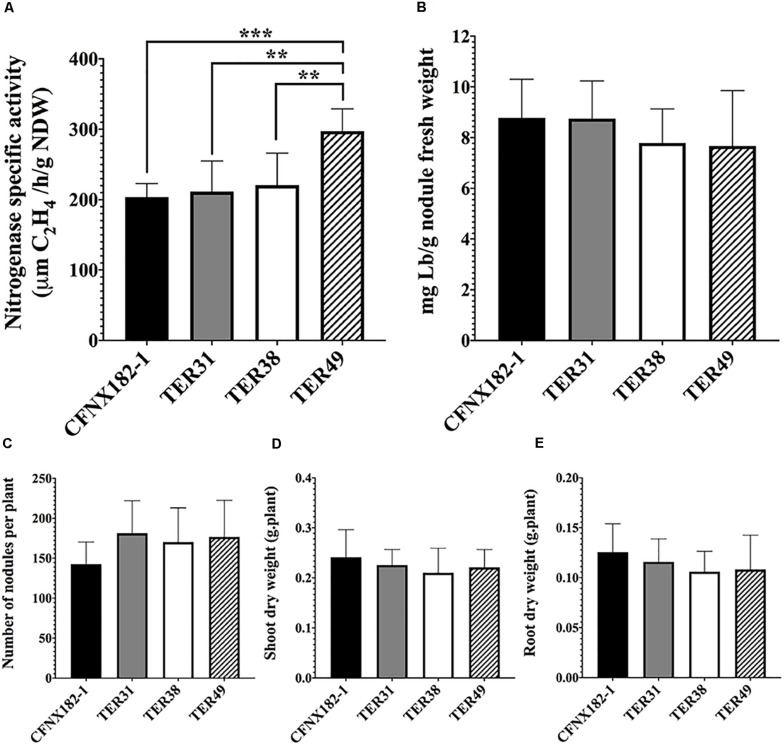
Symbiotic performance of TER strains with *P. vulgaris*. Bean plants were inoculated with the different strains, after 21 dpi symbiotic performance was analyzed by: **(A)** nitrogenase-specific activity by ARA, **(B)** leghemoglobin content, **(C)** number of nodules per plant, **(D)** shoot dry weight, and **(E)** root dry weight. Strains: CFNX182-1 (black bars), TER31 (gray bars), TER38 (white bars), and TER49 (striped bars). A *t-*test (*P* < 0.05), was used to determine statistically significant differences in the nitrogenase specific activity in **(A)**. 0.0021 (**), 0.0001 (****).

To determine if these nodules were able to fix nitrogen, we measured the nitrogenase-specific activity through an ARA assay and leghemoglobin content (see section “Materials and Methods”) of strains TER31, TER38, and TER49, as representative of alpha, beta, and gamma -proteobacteria. The results (Figure 3) showed that all strains did present nitrogenase activity; TER31 and TER38 nodules displayed nitrogenase-specific activity similar to that of the control, whereas a significantly higher activity was detected in TER49 nodules (Figure 3A). However, no significant differences in the leghemoglobin content were seen among the strains (Figure 3B). Nevertheless, the number of nodules produced by each strain (Figure 3C), and the weight of the dry root and shoot (Figures 3D,E) in TER samples were not significantly different from the control. The plants were also similar in size and color to the control strain (data not shown). In the experiments we employed hydroponic trays. This could be the reason for not detecting differences between the plants inoculated with the different strains.

These data indicate that TER31, TER38, and TER49 strains are able to induce the formation of N_2_-fixing nodules in *P. vulgaris* plants, suggesting that, at least *nod, fix* and *nif* genes from the pSym were transferred from CFNX182-1 to TER isolates.

### Visualization of Transconjugants on the Root Surface, in Infection Threads and in Nodules

To analyze if the TER strains adhere to and infect *P*. *vulgaris* roots similar to *R*. *etli*, the root surface, the initial infection features and nodules were visualized using confocal microscopy in TER infected roots (21 dpi). The results showed that TER31, TER38 and TER49 are able to adhere to the root surface, although the density of the bacterial cells seemed to be lower than in roots inoculated with *R. etli* (Figures 2A,D,G,J). They were able to produce infection threads (Figures 2B,E,K). As in nodules generated by rhizobia, in nodules incited by TER strains the infection is concentrated in the central tissue, where infected cells are visualized (Figures 2C,F,I,L). Most of the nodules formed by the three TER strains were similar to those of the *R. etli* control strain (Figure 2C).

**FIGURE 4:**
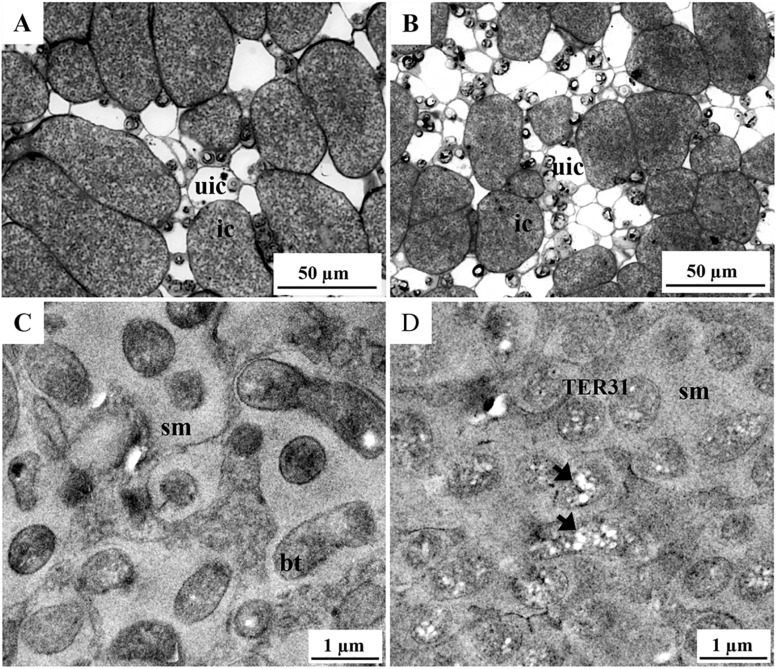
Histology and transmission electron microscopy of infected cells from nodules generated on roots inoculated with TER31 and CFNX182-1 strains. Semi-thin and ultra-thin sections of nodule central tissue (21 dpi) were analyzed by bright field **(A,B)** and transmission electron microscopy **(C,D)**. Samples: **(A,C)** nodules from roots inoculated with CFNX182-1 and **(B,D)** TER31. bt, bacteroids; ic, infected cells; sm, symbiosome matrix; TER31, intracellular TER31 bacteria; uic, uninfected cells; arrowheads, PHB-like storage material.

In an attempt to better describe the subcellular localization of TER31 in infected cells, we performed a comparative histological analysis of the nodule central tissue (Figures 4A,B). In toluidine blue stained sections of *R. etli*- infected nodules, the bacteria were easily distinguishable, infected cells had the typical pattern of dark dots and rods surrounded by a lightly stained area (Sánchez-López et al., 2011), an indicative of symbiosomes containing *R. etli* bacteroids (Figure 4A). In contrast, the bacterial population in TER31-infected cells displayed a poorly contrasted appearance, as if the dye barely reached the bacteria but stained their immediate environment (Figure 4B). A transmission electron microscopy examination lead us to determine that the intracellular TER31 bacteria are contained in a symbiosome-like structure. That structure is enriched in an electron-dense matrix that renders an inconspicuous contrast of TER31 features, other than the presence of abundant polyhydroxybutyrate (PHB)-like storage material when compared with CFNX182-1 (Figures 4C,D). It is known that differentiated rhizobia can form carbon storage compounds such as PHB and glycogen (Lodwig et al., 2005), although the symbiotic performance of rhizobia unable to synthesize PHB can be defective (*Ensifer meliloti*) or enhanced (*R. etli*). Also, it has been suggested that PHB may participate in stress protection, although the mechanisms are not clearly understood (Sun et al., 2019). Nevertheless, our data suggest that the genetic information of pRet42d present in the TER strains is sufficient to allow them to infect *P*. *vulgaris* plants, although the genomic background may be affecting the outcome of the symbiosis.

## Discussion

Endophytic bacteria have been shown to be able to colonize several tissues of the plant (Rosenblueth and Martínez-Romero, 2004; Turner et al., 2013). Specifically, in legumes, one of the most relevant groups of plants for human nutrition, endophytic bacteria have been isolated from nodules, roots, seeds and stems (Sturz et al., 1997; Rosenblueth and Martínez-Romero, 2004). These endophytic bacteria belong to many different genera from alpha-, beta-, and gamma-proteobacteria, and gram-positive bacteria (Kan et al., 2007; Moulin et al., 2015). In a comprehensive review about the nodule microbiome, Martínez-Hidalgo and Hirsch (2017) report the presence of many diverse non-rhizobia, some of them able to induce nitrogen-fixing nodulation. They speculate about the functions of the nodule-associated bacteria not involved in nodulation, suggesting that they have the potential to affect legume survival. In the first report of a gamma-proteobacteria that seems to generate effective nodules, Benhizia et al. (2004) found that the root nodules of three Mediterranean legume species contained diverse gamma-proteobacteria, including *Pantoea agglomerans, Enterobacter kobei, Enterobacter cloacae, Leclercia adecarboxylata, Escherichia vulneris*, and *Pseudomonas* sp. No evidence of any rhizobial-like sequence was found. Lima et al. (2009) using siratro as a trap plant, isolated efficient nodulating strains such as *Janthinobacterium* sp. (beta-proteobacterium) and *Stenotrophomonas* sp. (gamma-proteobacterium), from the soils of the Western Amazon region. It is possible that there are many more non-rhizobia able to nodulate, which may have been discarded as contaminants, due to the selection techniques used (Martínez-Hidalgo and Hirsch, 2017).

Although rhizobia that infect plant roots usually come from the rhizosphere, it has also been shown that some rhizobia species are found inside the legume’s seeds (Mora et al., 2014; López et al., 2018). Some of these rhizobia, after isolation, are also able to perform the complete infection process, attaching to the root hairs, generating infection threads and invading nodules, in the known symbiotic procedure.

In this work, we show that the pSym of *R. etli* CFN42 transfers to endophytic recipients inside nodules, allowing them to acquire new features. TER strains were isolated from nodules of bean seedlings inoculated exclusively with the labeled donor strain CFNX182-1, which contains a RFP marker in the chromosome and a GFP marker on the pSym, without an inoculated recipient strain. The putative transconjugants recovered from nodules were initially checked for presenting green and lacking red fluorescence and containing the Sp resistance associated to the GFP marker. Fifty-five strains showed these characteristics, they were further analyzed for presence of the GFP and symbiotic genes, using PCR. Only nine strains gave positive results, indicating that a high proportion of the putative transconjugants were false positives, probably due to endogenous fluorescence or Sp resistance. The genus of each of the nine TER strains that did show positive signals for the GFP and symbiotic genes was determined by sequencing PCR products obtained with primers from the 16S rRNA gene. The results showed that the transconjugants belonged to diverse genera, further indicating that nodules constitute an environment where bacteria may interact with each other. Surely many of the genetic interactions are not fruitful, due to impossibility of conjugation, recombination, gene expression, or other factors. But those events that are successful may be conducive to the generation of variants with new features, providing them with an advantage for survival. We cannot disregard that non-cultivable bacteria could also receive the symbiotic genes. Analysis of the capacity to establish symbiosis with bean roots showed that only four of the nine TER strains were able to nodulate and fix nitrogen, however the microscopy analyses indicated differences with the nodules formed by the *R. etli* strain. This finding is not unexpected, as the genomic background also may affect the symbiotic features. The transfer of symbiotic genes to endophytes inside the nodules, irrespective of the genus of the recipients, could be one of the mechanisms through which plant bacteria interactions have evolved, generating new variants with better symbiotic capacities. This suggestion agrees with data reported in the review by Andrews et al. (2018), where they found phylogenetic incongruence between core and symbiosis genes for strains of 14 out of the 15 currently accepted genera of rhizobia, concluding that transfer of symbiosis genes is not restricted by geography or specific genera, and may allow bacteria adapted to local soil conditions to acquire the ability to nodulate new hosts. Also, in our previous work (Cervantes et al., 2011) we found a very nice example of a conjugative non-symbiotic plasmid (pSfr64a) from *Sinorhizobium fredii* GR64, a strain isolated from bean nodules in Granada, Spain. This plasmid carries large segments of different evolutionary origins, 23% of the plasmid is highly similar to sequences of the chromosome of *S. fredii* NGR234, 22% showed similarity to pRet42a and 31% to the pSym (pRet42d) from *R. etli* CFN42.

Since the information that many pSyms are transmissible was available in the literature, researchers have used this to introduce symbiotic information into different strains, trying to generate strains able to effectively nodulate other plant hosts. The effectivity of transconjugants generated is usually lower than that of the parental (Martinez et al., 1987; Rogel et al., 2001). We think that our results regarding the isolation of effective transconjugants are due to the approach we used, where we let the plant do the work and select the transconjugants. The fact that *P. vulgaris* is a promiscuous legume, able to be nodulated by many different strains may also have been a contributing factor (Wang et al., 2019). Surely the genetic background of the recipients is an important factor, for the outcome of the conjugation. Questions that remain for the future are the determination of how competitive the TER strains are in the presence of other nodulating strains, and the isolation of the putative recipients, which would be very helpful to determine the scope of possible transfers. Recently, Doin de Moura et al. (2020) introduced the symbiotic plasmid of *Cupriavidus taiwanensis* into the plant pathogen *Ralstonia solanacearum*, and repeatedly inoculated the *C. taiwanensis* host, *Mimosa pudica* with the transconjugant. This experimental evolution allowed for the selection of derivatives able to nodulate and improve efficiency after various passages through the plant. These results support our assumption that the plant constitutes an environment strongly affecting the bacterial diversification and evolution.

Also, there have been some interesting efforts trying to determine the minimal set of genes required for effective nodulation. A very elegant approach was employed by diCenzo et al. (2016). Using *Sinorhizobium meliloti* strain Rm2011, they transferred the region containing essential genes from the pSymB into the chromosome, this allowed them to obtain derivatives cured of pSymB, as well as of pSymA, without affecting growth fitness. They reintroduced the plasmids into the cured strain, recovering the wild-type phenotype for alfalfa nodulation. Finally, they identified single copy essential symbiotic genes, through the generation of deletion mutants in both plasmids. The single copy genes required for symbiosis were located in four regions, corresponding to 12% of pSymA and pSymB. This minimal set of symbiotic genes was identified in the *Sinorhizobium* chromosomal background. Would this minimal set function in the background of a non-rhizobia? Could we identify a minimal set of symbiosis genes for *Phaseolus vulgaris*? Which genes of the chromosomal background are required for the minimal set to function? Would these genes vary among strains from different genera? Answers to all these questions could impact on the possibility to employ synthetic biology to manipulate the process to obtain improved nitrogen fixation in soils.

The pSym is a large plasmid (371 Kb), however, we could not visualize a *bona fide* pSym in any of the TER strains recovered in Eckhardt type gels (data not shown). Nevertheless, *R. etli* pSym sequences were present in these strains, and their antibiotic resistance marker was stable. A possible explanation for these data is that the pSym sequences are integrated in the chromosome of the TER strains. The fact that the donor strain lacked plasmid pRet42a suggests that transfer of the pRet42d must have occurred through the transfer genes located in this plasmid, which are regulated by the RctA/RctB system. Conjugative transfer genes of the pSym have been shown to be repressed, until an unknown signal activates their expression (Sepúlveda et al., 2008; Nogales et al., 2013). Our results suggest that the nodule constitutes an environment where pSym transfer may be activated. Further work will be needed to determine the manner in which this transfer system is induced under symbiotic conditions.

## Data Availability Statement

The original contributions presented in the study are included in the article/Supplementary Materials, further inquiries can be directed to the corresponding author/s.

## Author Contributions

LB-V, DC, SR, LC-D, RS-L, and LC performed the experiments, participated in analysis, and interpretation of the data. LB-V, GT, and SB conceived the work, integrated the data, and drafted the manuscript. All authors contributed to the article and approved the submitted version.

## Conflict of Interest

The authors declare that the research was conducted in the absence of any commercial or financial relationships that could be construed as a potential conflict of interest.

## Supplementary Material

FIGURE S1 PCR amplifications of *R. etli* pSym markers in the TER strains. To determine the presence of markers of the *R. etli* pSym in the putative transconjugants of endophytic recipients, DNA was isolated from the different strains and used as template to produce PCR products for *nodD1* **(A)**, *nodA* **(B)**, *fixNd* **(C)**, *nifH* **(D),** and GFP **(E)**, from the pSym of *R. etli* CFNX182-1. The lane labeled 182-1 corresponds to strain CFNX182-1, MWM – molecular weight markers.

FIGURE S2 Maximum-likelihood tree of 16S rRNA gene of the TER isolates from nodules of *Phaseolus vulgaris*. The tree shows the phylogenetic association of TER isolates with strains of different genera (reference type strains) belonging to Proteobacteria and Firmicutes phyla from according to their 16S rRNA gene sequences. The tree was built under the GTR model and rooted at the midpoint. The bar indicates the number of expected substitutions per site under this model. The bootstrap support value (100 replicates) of the branches of tree is shown in each node.

FIGURE S3 Stability of pSym-associated antibiotic resistance markers in TER strains. The different TER strains were inoculated into antibiotic-free media, and samples were taken at inoculation (T_0_) and after 72 h of incubation (T72), to determine the loss of antibiotic resistance, by quantifying the populations in the presence and absence of antibiotic. Each strain was compared to the control strain CFNX182-1. **(A)** TER31, **(B)** TER38, and **(C)** TER49. CFNX182-1 with antibiotic (black bars), CFNX182-1 without antibiotic (gray bars), TER strains without antibiotic (white bars), TER strains with antibiotic (striped bars).
