# Supplementary material for: Transfer of the Symbiotic Plasmid of Rhizobium etli CFN42 to Endophytic Bacteria Inside Nodules
Source: Front Microbiol. 2020 Jul 29;11:1752. doi: 10.3389/fmicb.2020.01752 (PMC7403402; doi:10.3389/fmicb.2020.01752)
Supplement: FIGURE S1 — PCR amplifications of R. etli pSym markers in the TER strains. To determine the presence of markers of the R. etli pSym in the putative transconjugants of endophytic recipients, DNA was isolated from the different strains and used as template to produce PCR products for nodD1 (A), nodA (B), fixNd (C), nifH (D), and GFP (E), from the pSym of R. etli CFNX182-1. The lane labeled 182-1 corresponds to strain CFNX182-1, MWM – molecular weight markers. [file Image_1.pdf]

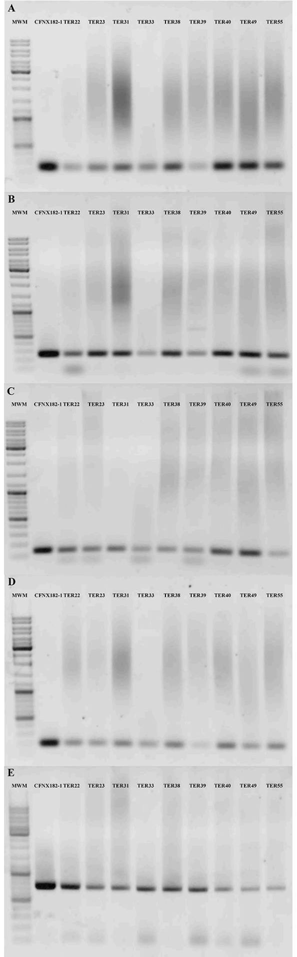

FIGURE S1. PCR amplifications of *R. etli* pSym markers in the TER strains.

To determine the presence of markers of the *R. etli* pSym in the putative transconjugants of endophytic recipients, DNA was isolated from the different strains and used as template to produce PCR products for *nodD1* (**A**), *nodA* (**B**), *fixNd* (**C**), *nifH* (**D**), and GFP (**E**), from the pSym of R. etli CFNX182-1. The lane labeled 182-1 corresponds to strain CFNX182-1, MWM – molecular weight markers.
