# Supplementary material for: Transfer of the Symbiotic Plasmid of Rhizobium etli CFN42 to Endophytic Bacteria Inside Nodules
Source: Front Microbiol. 2020 Jul 29;11:1752. doi: 10.3389/fmicb.2020.01752 (PMC7403402; doi:10.3389/fmicb.2020.01752)
Supplement: FIGURE S2 — Maximum-likelihood tree of 16S rRNA gene of the TER isolates from nodules of Phaseolus vulgaris. The tree shows the phylogenetic association of TER isolates with strains of different genera (reference type strains) belonging to Proteobacteria and Firmicutes phyla from according to their 16S rRNA gene sequences. The tree was built under the GTR model and rooted at the midpoint. The bar indicates the number of expected substitutions per site under this model. The bootstrap support value (100 replicates) of the branches of tree is shown in each node. [file Image_2.pdf]

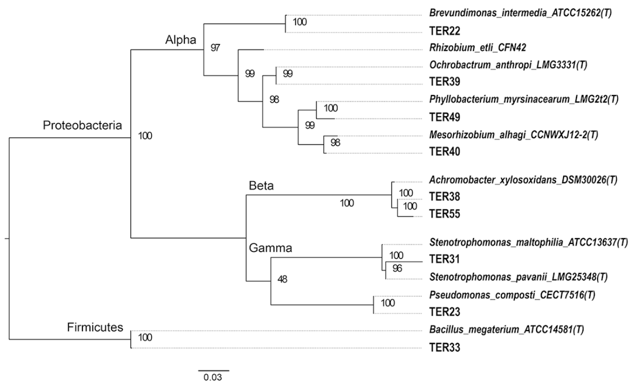

FIGURE S2. Maximum-likelihood tree of 16S rRNA gene of the TER isolates from nodules of *Phaseolus vulgaris*. The tree shows the phylogenetic association of TER isolates with strains of different genera (reference type strains) belonging to Proteobacteria and Firmicutes phyla from according to their 16S rRNA gene sequences. The tree was built under the GTR model and rooted at the midpoint. The bar indicates the number of expected substitutions per site under this model. The bootstrap support value (100 replicates) of the branches of tree is shown in each node.
