# Supplementary material for: Transfer of the Symbiotic Plasmid of Rhizobium etli CFN42 to Endophytic Bacteria Inside Nodules
Source: Front Microbiol. 2020 Jul 29;11:1752. doi: 10.3389/fmicb.2020.01752 (PMC7403402; doi:10.3389/fmicb.2020.01752)
Supplement: FIGURE S3 — Stability of pSym-associated antibiotic resistance markers in TER strains. The different TER strains were inoculated into antibiotic-free media, and samples were taken at inoculation (T0) and after 72 h of incubation (T72), to determine the loss of antibiotic resistance, by quantifying the populations in the presence and absence of antibiotic. Each strain was compared to the control strain CFNX182-1. (A) TER31, (B) TER38, and (C) TER49. CFNX182-1 with antibiotic (black bars), CFNX182-1 without antibiotic (gray bars), TER strains without antibiotic (white bars), TER strains with antibiotic (striped bars). [file Image_3.pdf]

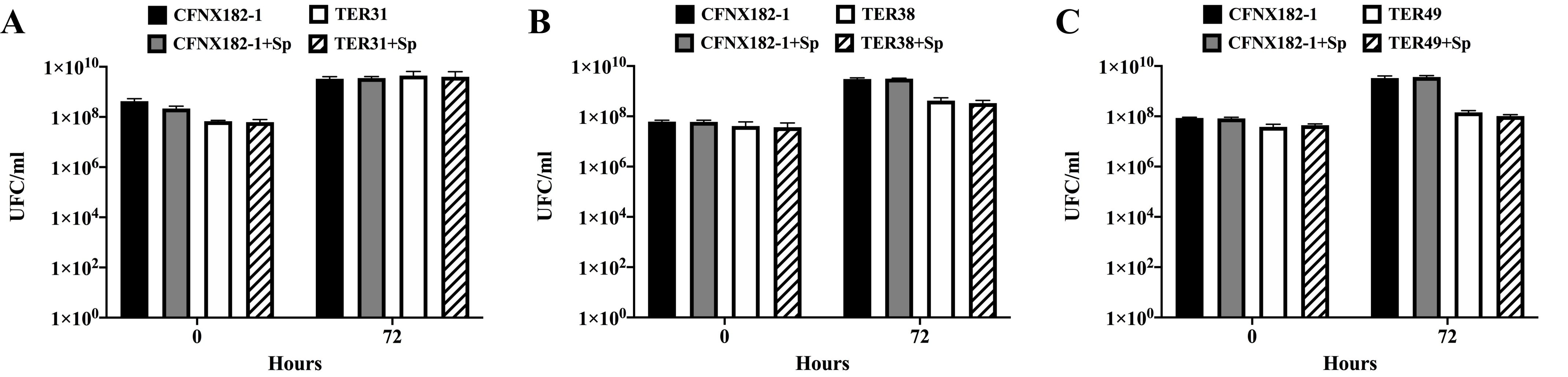

FIGURE S3. Stability of pSym-associated antibiotic resistance markers in TER strains. The different TER strains were inoculated into antibiotic-free media, and samples were taken at inoculation (T0) and after 72 h of incubation (T72), to determine the loss of antibiotic resistance, by quantifying the populations in the presence and absence of antibiotic. Each strain was compared to the control strain CFNX182-1. (**A**) TER31, (**B**) TER38, and (**C**) TER49. CFNX182-1 with antibiotic (black bars), CFNX182-1 without antibiotic (gray bars), TER strains without antibiotic (white bars), TER strains with antibiotic (striped bars).
