# Supplementary material for: Transfer of the Symbiotic Plasmid of Rhizobium etli CFN42 to Endophytic Bacteria Inside Nodules
Source: Front Microbiol. 2020 Jul 29;11:1752. doi: 10.3389/fmicb.2020.01752 (PMC7403402; doi:10.3389/fmicb.2020.01752)
Supplement: Supplementary file 4 [file Table_1.DOCX]

**Table S1. Oligonucleotides used in this study.**

| **PCR Primers (5’ -> 3’).** |  | **References** |  |
| --- | --- | --- | --- |
| Up-*nodD*1 | 5’- TCTGCTTCACATCCAGGTTTCG -3’ | Rodríguez et al., 2020 submitted |  |
| Lw-*nodD*1 | 5’- CGACATGAGCAATTCCGGAAAG -3’ | Rodríguez et al., 2020 submitted |  |
| Up-*nodA* | 5’- CTGGAGCTTACAACGCCCTT -3’ | Rodríguez et al., 2020 submitted |  |
| Lw-*nodA* | 5’- CAAACCGAACGCCAAGTTGC -3’ | Rodríguez et al., 2020 submitted |  |
| Up-*fixNd* | 5’- GCGCACGATCATCTGTTT -3’ | Rodríguez et al., 2020 submitted |  |
| Lw-*fixNd* | 5’- CGTCGCAATAGCCCGTAA -3’ | Rodríguez et al., 2020 submitted |  |
| Up-*nifH* | 5’- AATCGCATTTTACGGCAAAG -3’ | Rodríguez et al., 2020 submitted |  |
| Lw1-*nifH* | 5’- AAGGTCTTCCACCGAACCTT -3’ | Rodríguez et al., 2020 submitted |  |
| Up-GFP-R1 | 5’- TTTTGAATTCATTAAAGAGGAGAAATTAAGCATGCG -3’ | (Bañuelos-Vazquez et al., 2019) |  |
| Lw-GFP-H3 | 5’- CCAAGCTCAGCTAATTAAGCTTATTTG -3’ | (Bañuelos-Vazquez et al., 2019) |  |
| fD1 | 5’- CCGAATTCGTCGACAACAGAGTTTGATCCTGGCTCAG -3’ | (Weisburg et al., 1991) |  |
| rD1 | 5’- CCCGGGATCCAAGCTTAAGGAGGTGATCCAGCC -3’ | (Weisburg et al., 1991) |  |
| p42d_left_in | 5’- CCCATGCGGCTCACTC -3’ | This work |  |
| p42d_left_out | 5’- CGGGTGATCGGCTTTG -3’ | This work |  |
| p42d_right_in | 5’- CTGCCTCCGGGGTCTC -3’ | This work |  |
| p42d_right_out | 5’- TGCCCGGTGCTCTTTC -3’ | This work |  |
| CasNot-Ter-out | 5’- CCGTCTGTGATGGCTTCC -3’ | This work |  |
| Pneo-out | 5’- GGCAAGAAAGCCATCCAG -3’ | This work |  |
